# Modified Cow’s Milk as a Substitute Food in Infant Feeding

**Published:** 1912-12

**Authors:** 


### Modified Cow's Milk as a Substitute Food in Infant Feeding

The subject of modified milk as a substitute food for infant feeding has been studied from many points of view, but two facts are being recognized, more and more, as of prime importance—first, that cow's milk is the most practicable substitute food for infants; and, second, that it is just as important that the physical characteristics of cow's milk be modified as to the proportions of its food elements.

It is along these lines that First Lieutenant W. E. Fitch of the Medical Reserve Corps, U. S. A., has written a most practical paper upon the subject of "Modified Cow's Milk as a Substitute Food in Infant Feeding," published in *Pediatrics* (October, 1912). He studies the comparative chemical composition of healthy woman's milk and cow's milk, the general availability of cow's milk as a substitute food, the physical and chemical differences between cow's milk and woman's milk and the modification of cow's milk with cereal decoctions.

He emphasizes the necessity of using pure cow's milk, not milk that has been pasteurized or sterilized, but fresh, wholesome milk from a healthy herd. We all recognize the fact that the milk offered for sale in the large cities is not as pure as it should be, but, under the active work of the boards of health and the medical profession, it is rapidly improving in quality. When procurable, certified milk should always be used.

Dr. Fitch points out the fact that the modification of cow's milk with a cereal is a mechanical one due to the gelatinized starch, which changes the hard curdling cow's milk into a soft curdling milk like human milk. The casein of cow's milk clots in hard, lumpy masses in the infant stomach, the digestive enzymes cannot get at it, and any means whereby we can break up the clot and make it more flocculent will increase the digestibility of the milk; and this can be done by the use of a properly prepared cereal decoction.

Not only do cereals modify the casein of cow's milk, but they also, through their gelatinized starch, facilitate the digestion of fats, by emulsifying the fats after proteid digestion in the stomach. This is important because, as Holt shows, the tendency today is to give a large percentage of fat, and the fats of cow's milk are more difficult to digest than the fats of human milk. With many infants it is often necessary to begin with an amount less than 2 per cent of fat, and rarely is it necessary to exceed 4 per cent. There are numerous healthy infants who cannot even digest 4 per cent of fat at any time, and many, during the hot weather, do better on a reduction to 3 or 3.5 per cent.

Theoretically, the child under six months, because of the deficiency of salivary and pancreatic secretions, is said to be incapable of digesting starches. Practically this is not true. Nearly every fluid in the human economy has a diastatic ferment and, as a matter of fact, the very young infant does digest starch. We have seen too many babies successfully fed on arrow root to deny this fact. The author quotes Finkelstein, of Berlin, whose experience and general sound judgment are respected by the leading pediatricians of the world, who is emphatic that very young children are capable of digesting starches, and quotes favorable published opinions by Jacobi, Epstein, Schmid, Minard, Keller, Newman, Heubner and others, while our own Kerley has conclusively shown by his experiments at the New York Infant Asylum that "there is no age limit for cooked starch feeding."

The addition of cereals to cow's milk is not only allowable, but is to be most warmly recommended, not only in older, but also in very young infants. The advantages of cereal modification, in addition to the readier digestion and gain in weight, are to be found in the finer subdivision of the casein in the stomach, in the emulsification of the fat, in the disappearance of soapy and dyspeptic stools, in the proteid-sparing power afforded by the cereals, and, finally, in the general increment of growth.

This is the experience of the leading pediatrists of the world. Not every infant, by any means, can take cow's milk, or ass's milk, or goat's milk; but that starch foods may be added with benefit to cow's milk in the majority of cases is established beyond all question, experimentally, chemically and clinically.

Dr. Fitch then considers the practical details of cereal modification, and gives formulas for milk mixtures, based on years of successful use. He gives, also, clinical reports upon a number of cases had with these formulas.

The article is an exceedingly clear and practical consideration of the much befuddled question of the modification of cow's milk for infant use; and, best of all, it contains usable information.

